# A Unique Presentation of Fahr's Syndrome Secondary to Hypoparathyroidism

**DOI:** 10.7759/cureus.16063

**Published:** 2021-06-30

**Authors:** Zaraq Rashid Khan, Waqar Waheed, Jawad Mabood, Asim Ali, Gulalai Burki

**Affiliations:** 1 Internal Medicine, Hayatabad Medical Complex Peshawar, Peshawar, PAK; 2 Internal Medicine, Mufti Mehmood Memorial Teaching Hospital, Dera Ismail Khan, PAK; 3 Internal Medicine, Khyber Medical University, Peshawar, PAK; 4 Internal Medicine, Hayatabal Medical Complex, Peshawar, PAK; 5 Gyanecology, Khyber Medical University, Peshawar, PAK

**Keywords:** fahr's syndrome, hypoparathyroidism, parkinson, thyroidectomy, aphasia

## Abstract

Fahr's syndrome is a rare condition characterized by deposition of bilateral symmetric calcium deposits in the basal ganglia and cerebellar region, leading to neurological and psychiatric sequelae. Herein we describe a case of a 62-year-old female presented with aphasia, bilateral lower limb rigidity, tremors, and gait disturbance. Her past medical history included thyroidectomy and radiation therapy 10 years back due to papillary carcinoma of the thyroid gland. On examination, she had poor speech, resting tremor, walking difficulty, and decreased power in all limbs with rigidity. Her Chvostek and Trousseau signs were positive. Serum investigations revealed hypocalcemia and low levels of parathyroid hormone and thyroid-stimulating hormone. Brain magnetic resonance imaging revealed calcified lesions in basal ganglia, thalami, and dentate nuclei. She was diagnosed with Fahr's syndrome due to hypoparathyroidism, and she was managed with calcium gluconate, vitamin D, salt-free albumin, and levodopa-carbidopa, improving her condition. The patient was then discharged on calcium gluconate, calcitriol, recombinant parathyroid hormone, and levodopa-carbidopa with follow-up.

## Introduction

Fahr’s syndrome is characterized by the deposition of bilateral symmetric calcium deposits in the brain, more commonly in the basal ganglia and cerebellar region, leading to neurological and psychiatric sequelae [1]. Fahr’s syndrome and Fahr’s disease both are different clinical entities. Although both clinical conditions may have similar signs and symptoms, there are clear differences regarding the etiology of the disease, location of the lesions, treatment, and prognosis [2]. Fahr’s syndrome is a rare condition with a prevalence of 1/1,000,000, and the total number of cases reported so far is less than 200 [3]. Fahr’s syndrome has varied clinical presentation ranging from motor symptoms including tremors, ataxia, rigidity, and aphasia to psychiatric manifestations such as hallucinations, delusions, and cognitive impairment [4]. Here we present a case of Fahr’s syndrome secondary to hypoparathyroidism. The purpose of this case report is to play our part in helping the global medical community for a better understanding of the etiology, symptoms, and even management of this disease, about which very little is known so far.

## Case presentation

A 62-year-old female was brought to the emergency department for complaints of aphasia, anorexia, and bilateral lower limb rigidity for the last month. In addition, she had tremors in both hands and gait disturbance. Her symptoms started gradually and worsened during the last week, especially her speech. Her past medical history included thyroidectomy and radiation therapy ten years back due to papillary carcinoma of the thyroid gland. After that, she was put on thyroxin, calcium, vitamin D supplements, and she was noncompliant with her medication for the last two years.

On clinical examination, she looked confused and anxious. She was afebrile, and her blood pressure was 110/80 mmHg, respiratory rate 21/minute, heart rate 79/minute, and oxygen saturation 96%. On neurological exam, she had an altered level of consciousness, poor speech following simple commands, and mild resting tremor in both hands without myoclonus. Her Glasgow Coma Scale (GCS) was 11/15. Her Chvostek and Trousseau signs were positive; however, she had no signs of meningeal irritation and neck stiffness. She had a power of 4/5 in upper limbs and 3/5 in lower limbs with rigidity and had difficulty walking. Reflexes in the limbs were normal, and planters were downgoing bilaterally. Abdominal examination revealed mild tenderness in the hypogastric region, and the rest of the review was unremarkable. Initial serum investigations revealed low levels of parathyroid hormone (1.2 pg/mL) and thyroid-stimulating hormone (0.098 mIU/L). The coagulation profile and blood glucose were within the normal range. The results of blood workup on day one and day six are shown in Table 1.

**Table 1:** The results of initial blood investigations.

Parameter	Day 1	Day 6	Reference range
Hemoglobin (mg/dL)	8.48	9	11.5-17.5
White blood cell (mm^3^)	14,300	11,121	4,000-11,000
Red blood cell (million cells/mm^3^)	3.42	3.43	4-6
Sodium (mmol/L)	130	135	135-150
Calcium (mg/dL)	3.7	5.0	8-10
Magnesium (mg/dL)	1.8	1.8	1.7-2.2
Potassium (mmol/L)	3.85	2.89	3.5-5.1
Chloride (mmol/L)	91	97	96-112
Phosphorus (mmol/L)	1.99	1.5	0.87-1.45
Serum albumin (g/dL)	1.7	2.1	3.5-5.5
Creatinine (mg/dL)	3.1	1.8	0.9-1.1
Blood urea nitrogen (mg/dL)	117	51	18-45
Alanine aminotransferase (IU/L)	11	12	10-50
Aspartate aminotransferase (IU/L)	25	27	8-35
Alkaline phosphatase (IU/L)	71	72	45-125
25-hydroxyvitamin D (pg/mL)	19.65	36.21	>30

**Figure 1:**
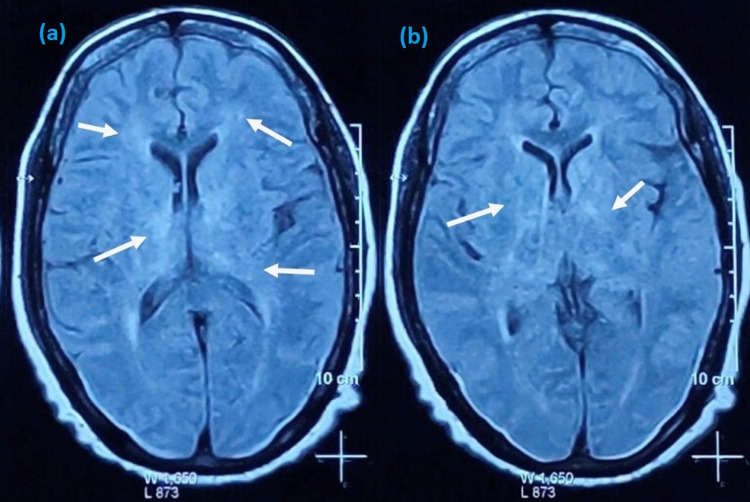
CT brain showing extensive calcifications in bilateral globus pallidi, thalami, and dentate and caudate nuclei (white arrows) in the axial plane (a, b). CT: computed tomography.

**Figure 2:**
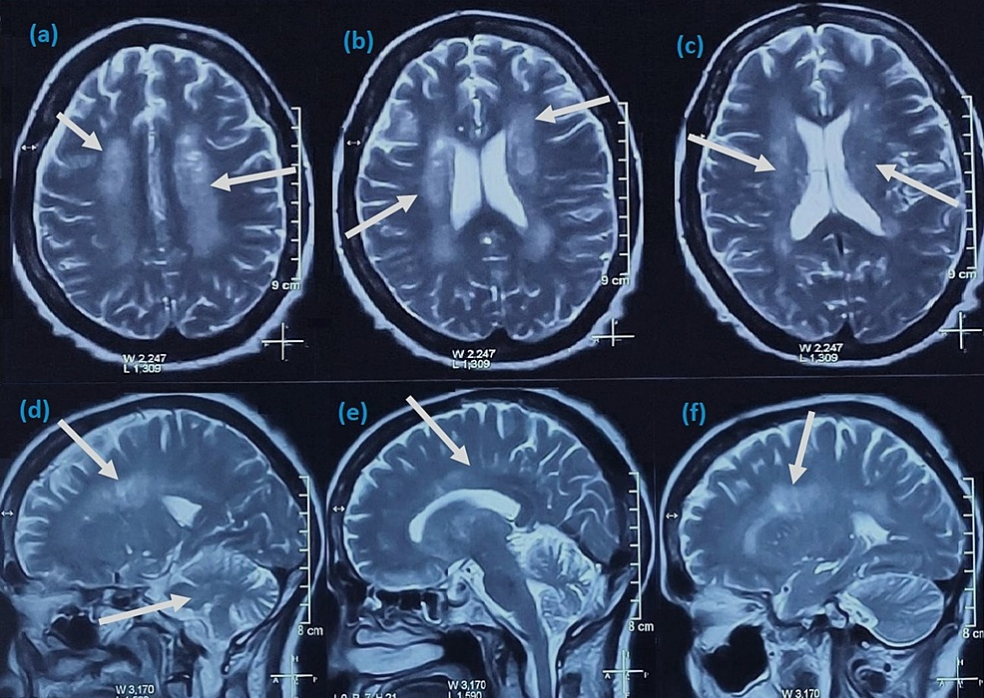
T-2-weighted MRI of the brain showing hyperintense signals (calcifications) bilaterally in periventricular white matter, internal capsule, thalami, basal ganglia, and cerebellum (white arrows) in axial (a-c) and sagittal (d-f) planes.

Computed tomography (CT) of the brain revealed calcifications in basal ganglia, dentate nuclei, and grey-white matter junctions in the axial plane (Figures 1a, 1b). Brain magnetic resonance imaging (MRI) revealed calcified lesions bilaterally in basal ganglia, thalami, dentate nuclei, anterior falx, and posterior dura, along with ischemic microvascular changes and lacunar infarcts in the internal capsule (Figures 2a-2f). Abdominal ultrasonography was unremarkable, and her infectious workup was negative for any organism. There were no remarkable changes in pituitary hormones or liver function tests. Cortisol rhythm, tumor markers, and antinuclear antibody spectrum were within normal limits. She was diagnosed with Fahr's syndrome due to secondary hypoparathyroidism.

Based on the investigation results, the patient was started on calcium gluconate, which was initially given intravenously to correct the low calcium level rapidly. When the patient was stable enough, oral calcium and vitamin D supplements were started, and salt-free albumin was also given. All these measures helped restore the serum calcium level. Corrected serum calcium levels significantly improved her speech and anorexia. Her muscle rigidity, however, only mildly enhanced. She was then started on levodopa-carbidopa combination based on the high suspicion she might have developed Parkinson's disease-like symptoms secondary to Fahr's syndrome. The patient's symptoms responded to levodopa-carbidopa, and there was a significant reduction in her rigidity on the fifth day of hospitalization. The patient was then discharged on calcium gluconate, calcitriol, recombinant parathyroid hormone, and levodopa-carbidopa with follow-up.

## Discussion

Fahr's syndrome is a neurodegenerative condition characterized by neuropsychiatric and neurological manifestations. Fahr's syndrome is rare and occurs in people of 40-60 years [3]. Only a small number of cases have been reported in the literature. Asokan et al. reported a case of Fahr's syndrome with speech difficulty as an initial manifestation [5]. Mahmood et al. reported a case of movement disorder in a patient with Fahr's syndrome due to hypoparathyroidism [6]. Zhou et al. also highlighted a case of Fahr's syndrome due to primary hypoparathyroidism presenting with movement disorders [7]. Similarly, our patient had Fahr's syndrome secondary to hypoparathyroidism manifesting as aphasia and parkinsonian features, rarely reported in the literature.

Multiple factors contribute to the pathogenesis of Fahr's syndrome. The common causes include endocrinopathies such as hypoparathyroidism, pseudohypoparathyroidism, hyperparathyroidism, vasculitis (systemic lupus erythematous), infections (human immunodeficiency virus, brucellosis), and other inherited disorders such as neuroferritinopathy [8]. All the patients have the same pattern of calcifications regardless of the cause of Fahr's syndrome, and calcifications are typically seen in basal ganglia, thalamus, subcortical white matter, cerebellum, and corona radiata [2]. It is believed that the calcification begins within the vessel wall, eventually extending to the neurons. In our patient, the leading cause of Fahr's syndrome was secondary hypoparathyroidism. Her condition improved significantly after the correction of serum calcium level and starting on levodopa-carbidopa.

Clinical presentation of Fahr's syndrome is variable, and many patients remain asymptomatic. Patients with severe form present in later stages with neuropsychiatric manifestations such as cognitive impairment, hallucinations, delusions, and neurological manifestations include dementia, aphasia, gait disturbance, movement disorders, and sensory changes [4]. Diagnosis of Fahr's syndrome is based on clinical presentation, serological studies, and imaging studies of the brain. Serum calcium, magnesium, phosphate, serum parathyroid, calcitonin, and vitamin D levels are recommended [8]. CT and MRI of the brain are the imaging of choice in patients with Fahr's syndrome [9]. Management of Fahr's syndrome involves symptomatic management and treatment of the underlying cause. Clonazepam is prescribed for dystonia, atypical antipsychotics for neuropsychiatric manifestations, and seizures are managed with antiepileptics [10,11].

## Conclusions

Although rare, Fahr’s syndrome should be kept in mind in all the cases of progressive movement disorders and neuropsychiatric disturbances in older age. Fahr’s syndrome should be suspected if the patient has hypoparathyroidism, worsening neurological symptoms, and symmetrical and abnormal basal ganglia calcifications on imaging. Laboratory tests are mandatory in such cases for prompt registration of possible metabolic abnormalities. Any suspected hypoparathyroidism should be treated timely in patients who underwent thyroidectomy, preventing calcification formation and progression of the disease. Timely recognition and management are recommended to prevent clinical manifestations and disease progression.
